# Assessing the impact of sewage and wastewater on antimicrobial resistance in nearshore Antarctic biofilms and sediments

**DOI:** 10.1186/s40793-025-00671-z

**Published:** 2025-01-20

**Authors:** Melody S Clark, Benjamin H Gregson, Carla Greco, Harisree Paramel Nair, Marlon Clark, Claire Evans, Kevin A. Hughes, Kudzai Hwengwere, Marcus Leung, Lloyd S Peck, Caray A. Walker, William Chow

**Affiliations:** 1 British Antarctic Survey Natural, Environment Research Council, High Cross, Madingley Road, Cambridge, CB3 0ET UK; 2 School of Life Sciences, Faculty of Science and Engineering, Anglia Ruskin University, East Road, Cambridge, CB1 1PT UK; 3 Basecamp Research Ltd, Unit 510 Clerkenwell Workshops, 27 Clerkenwell Close, London, EC1R 0AT UK; 4 Ocean Biogeosciences, National Oceanography Centre, European Way, Southampton, SO14 3ZH UK; 5 Marine Biology and Ecology Research Centre, School of Biological and Marine Sciences, University of Plymouth, Drake Circus, Plymouth, PL4 8AA UK

**Keywords:** Biofilm, Sediment, Nanopore, Bacteria, Drug class, Resistance mechanism

## Abstract

**Background:**

Despite being recognised as a global problem, our understanding of human-mediated antimicrobial resistance (AMR) spread to remote regions of the world is limited. Antarctica, often referred to as “the last great wilderness”, is experiencing increasing levels of human visitation through tourism and expansion of national scientific operations. Therefore, it is critical to assess the impact that these itinerant visitors have on the natural environment. This includes monitoring human-mediated AMR, particularly around population concentrations such as visitor sites and Antarctic research stations. This study takes a sequencing discovery-led approach to investigate levels and extent of AMR around the Rothera Research Station (operated by the UK) on the Antarctic Peninsula.

**Results:**

Amplicon sequencing of biofilms and sediments from the vicinity of Rothera Research Station revealed highly variable and diverse microbial communities. Analysis of AMR genes generated from long-reads Nanopore MinION sequencing showed similar site variability in both drug class and resistance mechanism. Thus, no site sampled was more or less diverse than the other, either in the biofilm or sediment samples. Levels of enteric bacteria in biofilm and sediment samples were low at all sites, even in biofilm samples taken from the station sewage treatment plant (STP). It would appear that incorporation of released enteric bacteria in wastewater into more established biofilms or associations with sediment was poor. This was likely due to the inactivation and vulnerability of these bacteria to the extreme environmental conditions in Antarctica.

**Conclusions:**

Our results suggest minimal effect of a strong feeder source (i.e. sewage effluent) on biofilm and sediment microbial community composition, with each site developing its unique niche community. The factors producing these niche communities need elucidation, alongside studies evaluating Antarctic microbial physiologies. Our data from cultivated bacteria show that they are highly resilient to different environmental conditions and are likely to thrive in a warmer world. Our data show that AMR in the Antarctic marine environment is far more complex than previously thought. Thus, more work is required to understand the true extent of the Antarctic microbiota biodiversity, their associated resistomes and the impact that human activities have on the Antarctic environment.

**Supplementary Information:**

The online version contains supplementary material available at 10.1186/s40793-025-00671-z.

## Introduction

Antimicrobial resistance (AMR) is recognised as being increasingly important in all continents of our planet except in Antarctica [1]. In comparison to the rest of the world, Antarctica is largely shielded from the current global spread of AMR by its remoteness. At 14.2 million square kilometres, Antarctica is the fifth largest continent, 40% larger than Europe or equivalent to twice the size of Australia or the size of the USA and Mexico combined. Despite its vast size, the maximum population during the summer months of December to February, peaks at around 5,000 people. This transient population primarily comprises research scientists and support personnel located predominantly in 76 permanent research stations run by national governmental operators across the continent and offshore islands, around half of which close during the winter [2]. A growing number of tourists visit the area, with 122,000 visiting the continent in 2023/24, mostly the Antarctic Peninsula aboard ship cruises [3, 4]. However, despite the overall low levels of human visitation to the continent, some sites are highly visited [5]. There is evidence that impacts resulting from local human activities are becoming more widespread, as levels and distribution of tourism, and national operator activity increase [6].

Several studies have identified the occurrence of “natural” AMR in Antarctica, likely due to strong competitive interactions for the limited resources in this extreme environment [7–9]. Nevertheless, any human contact can bring with it the risk of introduction of “non-native” AMR genes (i.e. bacterial genes which have evolved to act against human produced semi-synthetic and synthetic antibiotics) and the most obvious route for this is via sewage disposal [10]. Most Antarctic tourist ships remove sewage waste generated on board via deposition at appropriate port facilities outside Antarctica, but sewage disposal is more problematic for National Antarctic Programmes (NAPs) with land-based infrastructures, such as research stations. For the majority of NAPs, it is not financially viable to remove sewage waste from Antarctica, so at coastal Antarctic stations sewage waste is generally disposed of directly into the sea. The Protocol on Environmental Protection to the Antarctic Treaty (effective since 1998) states that all waste should be removed from Antarctica, except for sewage and domestic liquid waste (i.e. grey water). Sewage waste may be discharged directly into the sea from coastal locations, provided that conditions exist for initial dilution and rapid dispersal and that large quantities of sewage waste (i.e. that generated by c. 30 individuals or more) are macerated before disposal. A 2008 study identified that around one third of permanent research stations and over two thirds of summer-only research stations lacked any form of treatment facility [11]. Since then this situation has improved considerably, with data from 2022 showing that 69% of station have some level of treatment [12]. Several stations are also being modernised or constructed, so treatment standards may be further enhanced, albeit alongside a general increase in population numbers. However, circumstances can fluctuate. For example, although Rothera Research Station has had a sewage treatment plant since 2003, it has been repeatedly subject to operational and technical challenges and at the time of this study was largely not effective. The plant was designed to service a maximum of 110 people but has been overwhelmed due to design deficiencies and by the influx of building personnel brought in to undertake station redevelopment work, which has boosted summer station population numbers to ~ 160. Although the area covered by research stations in Antarctica is minute compared to the size of the continent and the surrounding seas, they comprise a significant proportion of ice-free coastal areas and according to a 2019 study, are disproportionately concentrated in some of the most sensitive environments [3].

Antarctic environments commonly experience low temperatures, which can substantially reduce enteric bacterial metabolic activity [13]. Nonetheless, bacteria commonly found in sewage can become sub-lethally injured when released into polar marine environments and enter a viable but non-culturable (VBNC) state allowing them to survive several weeks or more [14]. The release of such non-native microorganisms with mobile genetic elements can potentially transfer AMR genes to local bacterial and animal populations [15, 16]. Indeed, previous culture-based studies have shown release of non-native microorganisms, including *Escherichia coli*, *Clostridium perfringens* and *Enterobacter spp* into the water column and sediments from research station sewage [17, 18]. In addition, using a combination of culture- and molecular-based approaches human microbial contamination has been shown, in a few cases of marine invertebrates and mammals [19–22].

Antarctica is often referred to as the “pristine continent”, with AMR present to a level akin to the pre-antibiotic era or rather a “pre-widespread human use of antibiotics” state [23]. Currently there is no clear picture of “non-native” AMR contamination (and potential spread) in Antarctica, largely due to the limited number of studies and widely varying methodologies used [10]. Such data is urgently needed and will have significant policy implications for the nations that govern Antarctic through consensus via the annual Antarctic Treaty Consultative Meeting, particularly regarding environmental protection.

The impetus for our study came out of a review describing the current knowledge on AMR in Antarctica [10]. We have implemented some of the recommendations in that review, including full documentation of sites (including global navigation satellite system (GNSS) co-ordinates), antibiotic sensitivity testing according to EUCAST guidelines and full data disclosure. Using both culture-dependent and culture-independent (molecular) techniques (including Oxford Nanopore MinION technology, to evaluate the utility of direct on-site sequencing), we aimed to identify the extent of AMR in the marine environment in, and around, the Rothera Research Station sewage treatment plant (STP). We specifically chose to target biofilm and sediment samples as these represent more stable microbial populations rather than the more transient wastewater, which is rapidly diluted as it is expelled from the sewage treatment plant into the surrounding bay.

## Materials and methods

### Site description

**Fig. 1:**
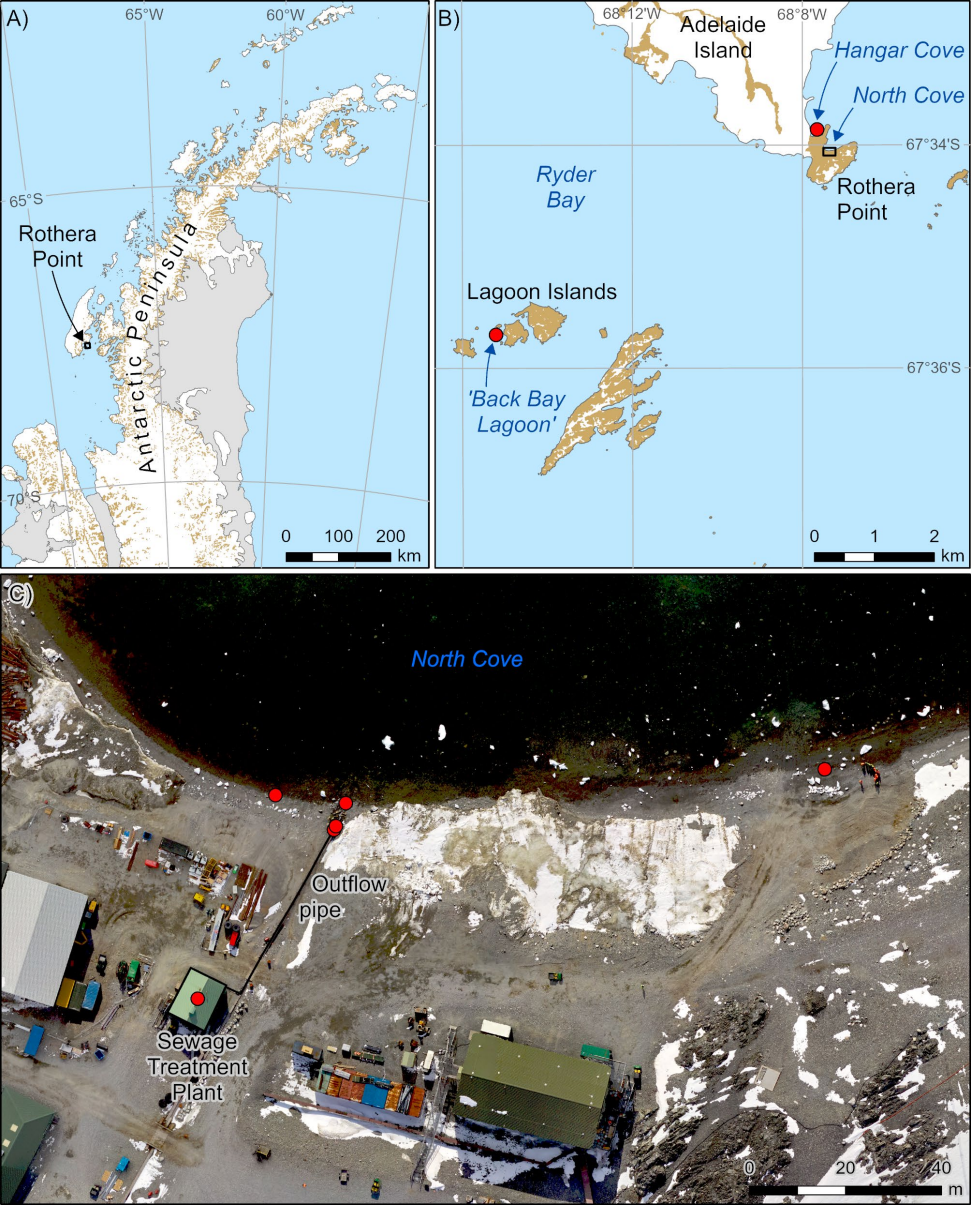
AMR sampling sites.** A**: Position of Rothera Point on the Antarctic Peninsula; **B**: Closer detail of Adelaide Island including Rothera Point and the two furthest sampling sites, Hangar Cove and “Back Bay” Lagoon; **C**: Aerial photography image of the sampling sites at Rothera around the sewage treatment plant in North Cove. Background image is taken from the Rothera Minimum Snow Cover Survey 2019 aerial image, British Antarctic Survey. GIS co-ordinates available in Additional File 1

**Fig. 2:**
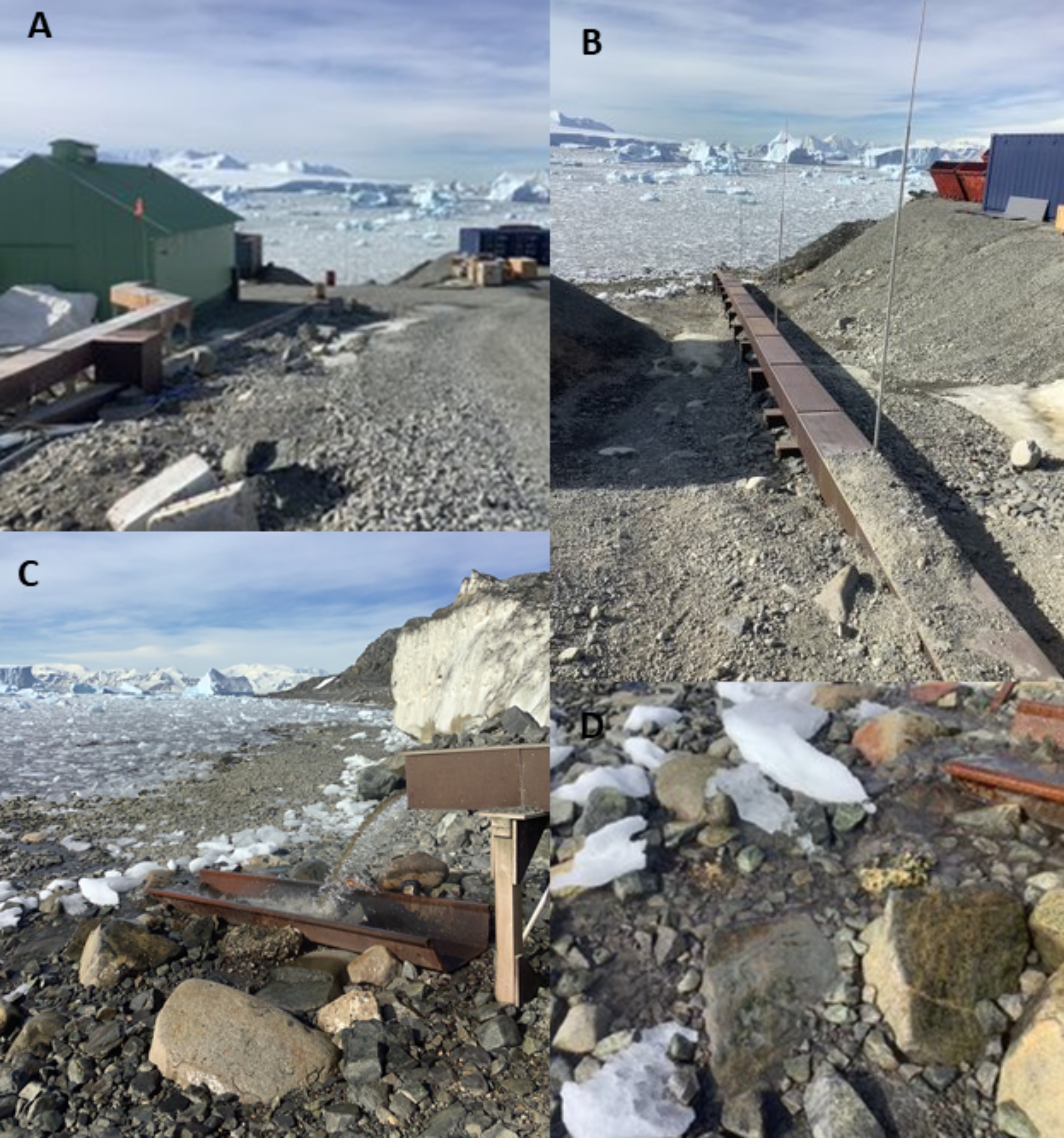
Sewage treatment plant (STP) and surroundings at Rothera**. A**: Main building housing the STP; **B**: The pipe (covered in wooden ducting) from the STP to the discharge point in North Cove; **C**: Effluent discharging into a trough in the intertidal region of North Cove. Note there are two pipes, one discharging grey water and the other discharging macerated waste from the STP. NB: Biofilm samples from the STP outflow were taken from the bacterial film lining the latter pipe; **D**: Intertidal zone of North Cove, just below the STP outflow. The “Just below the STP” biofilm samples were taken from rocks under the outflow

Rothera Research Station, operated by the UK national Antarctic programme, British Antarctic Survey (BAS), is situated on Rothera Point, Adelaide Island (67° 4’ 07” S, 68° 07’ 30” W) (Fig. 1). The station has been occupied continuously since it was founded in 1976 and now can accommodate up to 160 science and support personnel during the austral summer. During winter, occupancy levels are reduced to c. 20–30 personnel. Since 2003, sewage emptying into North Cove (Fig. 1c) has been processed through a treatment plant, but prior to this date, maceration was the only treatment [24]. To prevent damage to the outflow pipe by ice impacts, the sewage outfall pipe was installed at a height of ~ 1 m above the beach surface, close to the high tide mark, with boulders (up to 1 m across) placed on the beach around the outfall. The rest of the beach is predominantly characterized by compacted cobble pavements, with little sediment within the matrix (Fig. 2).

### Sampling procedures

Eight sites were sampled around Rothera Research Station (Fig. 1; GIS (Geographic Information System) co-ordinates in Additional File 1). Site abbreviations: BF = biofilm; Sed = sediment. STP: Sewage treatment plant; Out: Outflow pipe from the sewage treatment plant; JBSTP: sampled on rocks just below the sewage treatment plant outflow pipe; IT: intertidal; 100mE: 100 m east from the sewage treatment plant outflow pipe; 15mW: 15 m west from the sewage treatment plant outflow pipe; 15mWAn: anoxic sediment15m west from the sewage treatment plant outflow pipe; HC: Hangar Cove; BBL: Back Bay Lagoon Island. Five sites were sampled for biofilm and six sampled for sediment, with the intertidal and 100mE sites sampled for both biofilm and sediment. Each site was sampled in triplicate for biofilm and/or sediment. Biofilm, which appeared to be largely a mix of bacteria and algae, was swabbed from the STP tank, the outflow pipe and surface of rocks, while sediment was collected from intertidal sites and also diver-collected. Collections of sediment samples from Hangar Cove and Back Bay Lagoon (Fig. 1b) were hand collected at ~ 15 m depth by SCUBA divers in 2020. These samples were stored and returned to the UK at -20 °C for analysis. The other biofilm and intertidal sediment samples were collected from around the STP in 2022 (Figs. 1c and 2). Biofilm was collected on sterile cotton swabs and intertidal sediment was collected in sterile plastic 50 ml conical centrifuge tubes. Samples were returned to the Bonner Laboratory at Rothera Research Station and immediately used in either DNA extractions for metagenomics sequencing onsite using a MinION (Oxford Nanopore Technologies), or for the cultivation of marine bacteria, as described below. Additional samples were preserved at -20 °C to enable additional DNA extractions to be performed, if required.

### DNA extraction and sequencing

DNA was extracted from biofilm samples using the DNeasy^®^ PowerBiofilm^®^ kit (Qiagen) according to manufacturer’s instructions. DNA was initially extracted from all the sediment samples using the DNeasy^®^ PowerSoil^®^Pro kit (Qiagen). If insufficient DNA was obtained, further samples were extracted using the DNeasy^®^ PowerMax^®^ Soil kit (Qiagen) with downstream ethanol precipitation to concentrate the DNA. DNA was QC’ed on a Nanodrop One™ (ThermoScientific) and quantified on a Qubit 4 fluorometer (Invitrogen by ThermoFisher Scientific). DNA (400 ng at a concentration of 16ng/µl) from each site (in triplicate) was sent to Novogene (Cambridge, UK) for amplicon sequencing using the V3-V4 (341 F-806R) primer set [25]. DNA was sequenced on an Illumina NovaSeq 6000 with a 2 × 250 bp paired end chemistry. The DNAs from these sites were also subjected to long-read metagenomic sequencing on a MinION (Oxford Nanopore Technologies). Briefly, DNA samples undergoing MinION sequencing were purified and concentrated to at least 54 ng/µl using AMPure XP beads (Beckman-Coulter), with a DNA: AMPure bead ratio of 1:0.5 to size select for larger DNA fragments. A total of 400 ng of DNA was used in each sequencing reaction. Triplicate samples of DNA from each site were tagged using the Rapid Barcoding Kit (Oxford Nanopore Technologies) and sequenced on MinION flow cells (R9.4.1 version, Oxford Nanopore Technologies). Initial sequencing runs were performed at Rothera using remote MinKnow software (version 21.06.0). For consistency, all further sequencing runs performed in the UK were also run on this same software, except for the final run which, for software obsolescence reasons, was updated to MinKnow 22.12.7.

### Bioinformatic analysis of amplicon sequencing

DNA sequencing analysis was performed using the methods previously described [26, 27]. Amplicon sequencing reads were processed using the ‘*DADA2*’ (v1.8) pipeline [28], which can resolve exact biological sequences by assembling the reads into error-corrected amplicon sequence variants (ASVs). The pipeline consists of quality filtering, trimming, error correction and sample inference using the ‘*DADA2*’ algorithm at default parameters. Chimeras were removed and taxonomy was assigned to the resulting ASVs using the Ribosomal Database Project (RDP) naïve Bayesian classifier method [29] against the SILVA v138 database [30]. Non-locus-specific, or artefactual, ASVs were discarded prior to statistical analyses, along with any singletons and doubletons ASVs or those that had < 70% identity with any sequence in the database. There was an average of 1,575 ASVs per sample (ranging from 890 to 1,935). ASV counts were then normalised in the ‘*metagenomeSeq*’ package using cumulative-sum scaling [31]. The 16 S rRNA gene similarity between Sanger sequences for cultured representatives and ASV sequences was calculated using the ‘*Culturome*’ pipeline [32]. To examine the culture-dependent coverage, only ASVs that shared greater than 97% 16 S rRNA gene similarity with Sanger sequences for cultivated bacteria, were considered matched. *Statistical analysis*: All statistical analyses were carried out in R v4.1.2 [33]. All figures were generated using the ‘*ggplot2*’ [34] and ‘*cowplot*’ [35] packages. Data were first tested for normality with the Shapiro-Wilks test [36] and homogeneity with the Bartlett’s test [37]. Normally distributed data were tested for significance using ANOVAs, with *p*-values adjusted for multiple comparisons using the Benjamini-Hochberg procedure [38] followed by a Tukey’s HSD post hoc test [39] within the ‘*agricolae*’ package [40]. Non-normally distributed data were tested for significance using a Kruskal-Wallis test, with *p*-values adjusted for multiple comparisons with Bonferroni corrections [41] followed by Dunn’s post hoc test [42] within the ‘*FSA*’ package [43]. The top five most abundant bacterial phyla and genera across each sampling site were calculated and plotted from the normalised ASV count data in the ‘*phyloseq*’ package [44]. α-diversity metrics (ASV richness, Shannon Index and Inverse Simpson Index) were also calculated from the normalised ASV count data using ‘*phyloseq*’ [44]. β-diversity metrics were calculated using Bray-Curtis dissimilarity [45] in the ‘*vegan*’ package [46]. The resulting dissimilarity scores were visualised using non-metric multidimensional scaling (NMDS), to observe differences in the microbial community composition between sampling sites. Differences in community structure were further tested by permutational multivariate analysis of variance (PERMANOVA) [47] using the ‘*adonis2*’ function in ‘*vegan*’ [46]. A further pairwise PERMANOVA post hoc test (999 permutations) was performed in the ‘*pairwiseAdonis*’ package [48] to determine which communities at each sampling site were different to each other.

### Bioinformatic analysis of MinION data

The amount of sequence data obtained from each site was highly variable, therefore, triplicate samples from each site were pooled for analysis. To standardise basecalling, MinKnow 22.12.7 with Guppy 6.4.6 was run on data from all sites. A pipeline was written in Snakemake [49] using Singularity containers (version 3.8.6) [50] to run the assembly, polishing, and QC of the raw MinION data for each site. Data QC was performed using nanostat (version 1.6.0) [51]; assembly performed using metaflye (version 2.9.2) [52] (https://github.com/fenderglass/Flye) and polishing using Medaka (version 1.8.0) (https://github.com/nanoporetech/medaka). Assembly statistics were generated with Quast (version 5.2.0) [53]. Annotation of the assemblies was performed using the nf-core funcscan pipeline (version 1.1.1) (https://nf-co.re/funcscan). All programmes were run using default parameters unless stated. Funscan was run with parameters -- amp_skip_hmmsearch --amp_skip_amplify --bgc_skip_hmmsearch --arg_skip_deeparg. For the analysis of ARGs (antimicrobial resistance genes) we used the output from the hAMRonization program within funcscan. In particular, we concentrated on output data from the rgi fargene program with annotation from the CARD (Comprehensive Antibiotic Resistance Database) [54], as recommended [55]. The output from rgi fargene and CARD was collated for each site with regard to drug class and resistance mechanism of annotated ARGs. The ARGs were manually binned into the major drug classes as defined in [23, 56]. The β-lactam group included penam, penem, cephalosporin, carbapenem, cephamycin and monobactam. The MLS group included macrolides, lincosamides and streptogramin, whilst all tuberculosis (TB) drugs were included in the rifamycin group. There were no identifications of diterpenoids. Where two or more different drug classes were identified in the same contig, these were designated as multidrug resistant. There was an additional grouping for “other” (comprising any drugs not listed in the previous 15 categories). *Statistical analysis*: Differences in the number of ARGs detected between sampling sites, based on either drug class and resistance mechanism were tested using the statistical analysis methods described above (“Bioinformatic analysis of amplicon sequencing” section).

### Cultivation of bacteria

*Biofilm*: swabs were each added to a tube containing 2 ml of sterile phosphate-buffered saline (PBS) and vortexed to disperse the bacteria into the saline solution. Biofilm/saline solution (500 µl) was plated directly onto agar plates made from Marine Broth 2216 (Merck); R2A (Merck) and a 50% dilution of R2A. Further subculturing of the bacteria was carried out using 1:50 and 1:100 dilutions (in sterile saline) of the original biofilm/agar solution. Any remaining biofilm/saline solution was preserved as a 50% glycerol solution and stored at -20 °C and returned to the UK. The agar plates were incubated at 4 °C for ~ 4 months, whilst the samples were being returned to the UK by the *RRS Ernest Shackleton*. On return to the UK, to identify a unique set of marine cultivated bacteria from the biofilm samples, bacterial growth on the agar plates was examined carefully and morphologically different colonies were purified by re-streaking on fresh plates, as described above. The different strains of bacteria were defined by colour, morphology and growth pattern on different media. Strains were taxonomically identified via molecular barcoding using the V3-V4 primers (V3-341 F: CCTAYGGGRBGCASCAG; V4-806R: GGACTACNNGGGTATCTAAT) that produced an amplicon of ~ 430 bp [25]. Sanger sequencing of barcodes was performed by Source Bioscience, UK. Putative identification of species was performed using Blast sequence similarity searching, with designations only possible to genus level, due to the paucity of Antarctic sequences in the public databases. Within each assigned class/genus, multiple sequence alignments were performed in Geneious version 2022.2.2 to eliminate duplicate clones. Thermal tolerances of cultivated clones were determined either using liquid media (marine broth) (Hwengwere, unpublished data) or direct plating onto R2A media with incubations at 4 °C (control plate), 22 °C, 28 °C and 35 °C. Growth was assessed after 7 d and 11 d at all temperatures. *Enteric bacteria (E. coli)*: All work was carried out in the containment level 2 facility at Anglia Ruskin University. The preserved biofilm/saline samples (preserved in 50% glycerol and stored at -20 °C) were also used to screen for enteric bacteria. Using selective media, Eosin Methylene Blue Agar (EMBA), bacteria were plated directly onto the agar prior to overnight incubation at 37 °C. Colonies that were either purple with a green metallic sheen or a purple mucoid were purified using Brain Heart Infusion Agar and were taxonomically identified using the molecular methods described above.

*Antimicrobial resistance screening*: Antimicrobial susceptibility of both the cultivated marine and enteric bacteria were determined by the standard disc diffusion method. Commercial antibiotic discs (Oxoid, UK) representing multiple antibiotic classes were selected to screen the enteric isolates. Marine isolates were screened in R2A agar using the following 12 discs: Kanamycin (30 µg), Erythromycin (10 µg), Tetracycline (30 µg), Gentamicin (10 µg), Ciprofloxacin (5 µg), Pefloxacin (5 µg), Levofloxacin (5 µg), Cefepime (30 µg), Ceftazidime (10 µg), Cefotaxime(5 µg), Meropenem (10 µg) and Chloramphenicol (30 µg). Environmental isolates do not fall under European Committee on Antimicrobial Susceptibility Testing (EUCAST) guidelines (EUCAST.org), therefore, bacteria were classified as being resistant when the zone of inhibition had a diameter of less than or equal to 10 mm. Enteric isolates were incubated overnight at 37 °C, whereas the biofilm isolates were incubated at 25 °C for 3 d. The isolates were grown on Muller Hinton agar, as described in EUCAST guidelines, using the following 10 discs: Gentamicin (10 µg), Ciprofloxacin (5 µg), Pefloxacin (5 µg), Levofloxacin (5 µg), Ampicillin (10 µg), Cefepime (30 µg), Ceftazidime (10 µg), Cefotaxime (5 µg), Meropenem (10 µg) and Chloramphenicol (30 µg).

## Results

### Amplicon sequencing

**Fig. 3:**
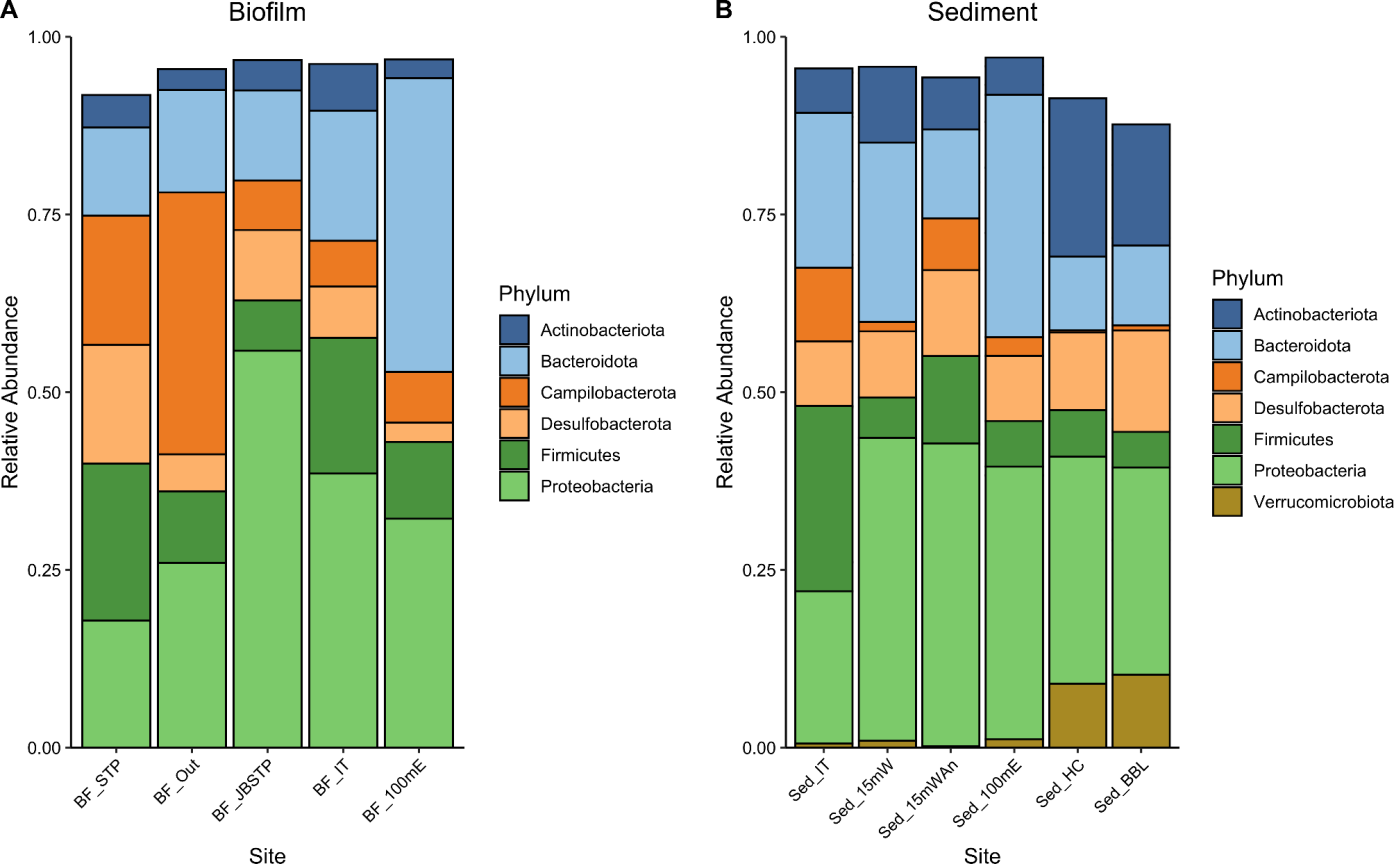
Phyla plot: Showing main Phyla in (**A**) biofilm samples and (**B**) sediment samples. Site abbreviations: BF = biofilm; Sed = sediment. STP: Sewage treatment plant; Out: Outflow pipe from the sewage treatment plant; JBSTP: sampled on rocks just below the sewage treatment plant outflow pipe; IT: intertidal; 100mE: 100 m east from the sewage treatment plant outflow pipe; 15mW: 15 m west from the sewage treatment plant outflow pipe; 15mWAn: anoxic sediment15m west from the sewage treatment plant outflow pipe; HC: Hangar Cove; BBL: Back Bay Lagoon Island (see Fig. 1 for map)

**Fig. 4:**
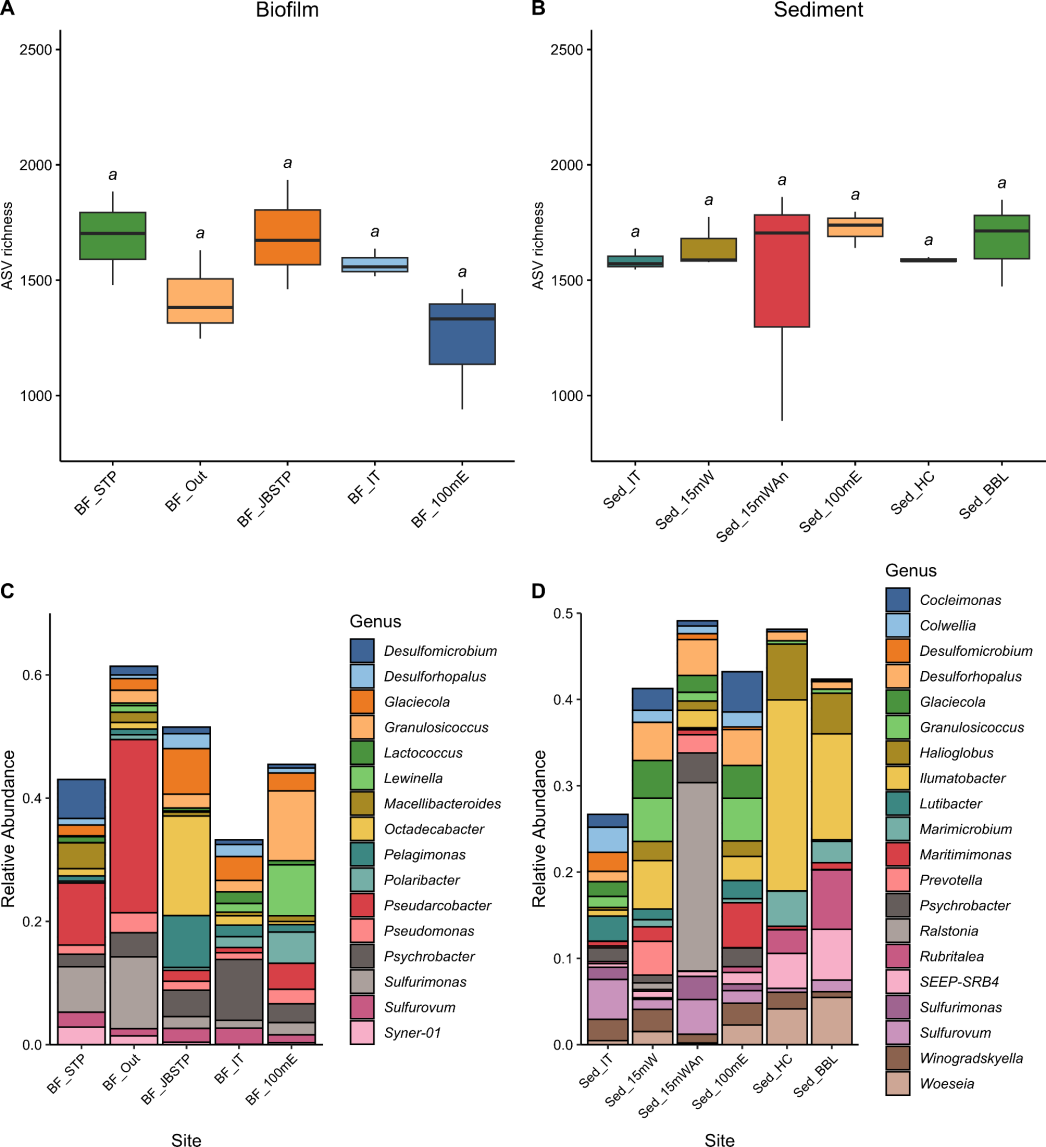
Alpha diversity plot. (**A**) ASV richness of biofilm samples; (**B**) ASV richness of sediment samples; (**C**) Relative abundance of different genera in biofilm samples; (**D**) Relative abundance of different genera in sediment samples. See Fig. 3 for site abbreviations

These sequencing data were used to conduct an initial survey of microbial biodiversity at each site and evaluate if they differed in community composition. These data revealed 6–7 main phyla of bacteria across the eight sites (Fig. 3). The highest relative abundance of Proteobacteria was in the biofilm samples collected just below the outflow pipe (BF_JBSTP) (55.84%) (Fig. 3A). *Actinobacteriota* had the highest relative abundance in sediment samples collected furthest from the sewage treatment plant in Hangar Cove (Sed_HC) and Back Bay Lagoon (Sed_BBL) (22.26% and 17.05%, respectively). This was also the case for *Verrucomicrobiota* (9.00% and 10.26%) (Fig. 3B), which were almost absent from the biofilm samples). At the phylum level *Campilobacterota* dominated the biofilm samples collected at the end of the outflow pipe (BF_Out) (relative abundance − 36.84%), whereas it only made up 7.13% in the BF_100mE samples, which were collected 100 m away from the source site. At the genus level most of the Campilobacterota in the biofilm film samples from the STP outflow were members of the *Pseudarcobacter* genus (relative abundance – 28.10%), which are typically found in high abundance in or near sewage or wastewater treatment plants (Venâncio et al. 2022) (Fig. 4). At the phyla level, *Bacteroidota* dominated the more distant biofilm (BF_100mE) samples (41.35%) and were much lower in and around the outflow pipe (BF_STP- 12.42%, BF_Out- 14.39%, BF_JBSTP- 12.69%) (Fig. 3A). Members included *Lewinella* sp. which had a relative abundance of 8.26% when sampled from 100 m east of the outfall, but in comparison had < 1% relative abundance in and around the outflow pipe (Fig. 4A). Overall, there was no significant difference in any of the alpha diversity metrics including ASV richness (Biofilm, ANOVA, F = 2.566, *P* = 0.103; Sediment, Kruskal-Wallis, χ^2^ = 4.0877, *P* = 0.5369), Shannon Index (Biofilm, ANOVA, F = 2.821, *P* = 0.084; Sediment, Kruskal-Wallis, χ^2^ = 5.1637, *P* = 0.3962) and Inverse Simpson Index (Biofilm, ANOVA, F = 2.96, *P* = 0.075; Sediment, Kruskal-Wallis, χ^2^ = 7.339, *P* = 0.197). Therefore, no site was more or less diverse than any other site either in the biofilm or sediment samples (Fig. 4A, B). There was also no significant difference in PERMANOVA results-based Bray-Curtis dissimilarity meaning microbial community composition overall was not different to each other and the communities were very homogenous (Biofilm, PERMANOVA, *R*^*2*^ = 0.286, F = 1, *P* = 1; Sediment, PERMANOVA, *R*^*2*^ = 0.29412, F = 1, *P* = 1).

### Cultivated biofilm bacteria

A panel of 39 seemingly morphologically different bacteria were cultivated and isolated from the biofilm samples. This panel was reduced to a clone bank of 21 non-redundant species, as defined by V3-V4 amplicon sequencing and multiple sequence alignments (Table 1, Additional File 2). The largest class comprised *Gammaproteobacteria* with 13 clones from 4 genera (*Psychrobacter* (5 isolates), *Pseudomonas* (4 isolates), *Shewanella* (3 isolates) and a single representative from the *Rheinheimera* genus). There were additional representatives from the *Flavobacteriia* (3 isolates), two isolates from each of the *Actinomycetes* and *Bacilli*, with a single representative of the *Cytophagia* (Table 1). Within each genus with multiple members, sequence identity varied from 94.2 − 99.3% across the ~ 430 bp amplicon (Additional File 2). Since these cultivated bacteria were identified via molecular barcoding using the same V3-V4 primers as used in the amplicon sequencing (detailed above), their abundances could be characterised within the amplicon sequencing data. These 21 bacteria were present at low numbers (i.e., less than 1% of relative abundance), sharing > 97% 16 S rRNA gene similarity to 55,329 ASVs in the amplicon data. These ASVs accounted for an average of 5,015 counts per sample. (Additional File 3). These cultivated bacterial strains were all europsychrophilical strains, surviving to at least 22 °C with 4 of the strains growing well at 35 °C (Table 1).

**Table 1:** Identities of cultivated bacteria from biofilm samples with experimentally derived temperature tolerances

Identifier	Class	Genus	Clone designation in this paper*	NCBI accession no	Temperature tolerance °C
BF1	Gammaproteobacteria	*Pseudomonas*	*Pseudomonas sp. A*	OR221159	28
BF2	Gammaproteobacteria	*Rheinheimera*	*Rheinheimera sp.*	OR221160	28*
BF3	Gammaproteobacteria	*Psychrobacter*	*Psychrobacter sp. A*	OR221161	28
BF4	Bacilli	*Trichococcus*	*Trichococcus sp.*	OR221162	35**
BF5	Gammaproteobacteria	*Psychrobacter*	*Psychrobacter sp. B*	OR221163	22
BF6	Gammaproteobacteria	*Pseudomonas*	*Pseudomonas sp. B*	OR221164	28**
BF8	Gammaproteobacteria	*Psychrobacter*	*Psychrobacter sp. C*	OR221165	28**
BF10	Gammaproteobacteria	*Pseudomonas*	*Pseudomonas sp. C*	OR221166	35**
BF13	Gammaproteobacteria	*Psychrobacter*	*Psychrobacter sp. D*	OR221167	28
BF14	Gammaproteobacteria	*Psychrobacter*	*Psychrobacter sp. E*	OR221168	35
BF15	Flavobacteriia	*Flavobacterium*	*Flavobacterium sp. C*	OR221169	22
BF17	Actinomycetes	*Arthrobacter*	*Arthrobacter sp.*	OR221170	28
BF18	Actinomycetes	*Brachybacterium*	*Brachybacterium sp.*	OR221171	28
BF20	Bacilli	*Planococcus*	*Planococcus sp.*	OR221172	35**
BF21	Cytophagia	*Algoriphagus*	*Algoriphagus sp.*	OR221173	28
BF23	Gammproteobacteria	*Pseudomonas*	*Pseudomonas sp. D*	OR221174	28
BF25	Flavobacteriia	*Flavobacterium*	*Flavobacterium sp. A*	OR221175	28
BF26	Gammaproteobacteria	*Shewanella*	*Shewanella sp. A*	OR221176	28
BF28	Flavobacteriia	*Flavobacterium*	*Flavobacterium sp. B*	OR221177	22
BF34	Gammaproteobacteria	*Shewanella*	*Shewanella sp. B*	OR221178	22
BF35	Gammaproteobacteria	*Shewanella*	*Shewanella sp. C*	OR221179	22

*Clone sequences were submitted to NCBI BankIt without the species designation of A, B, C etc. These are used within this paper to facilitate identification between different barcodes from the same genus

**Data from Hwengewere (unpublished)

### Cultivated enteric bacteria

A selection of 15 morphologically distinct enteric isolates were cultivated from biofilm samples. Of these, seven isolates were identified as being *E. coli*, four were *Klebsiella pneumoniae* and the remaining were comprised of *Enterobacter*, *Citrobacter* and *Raoultella.* Enterobacteriaceae were detected at low relative abundances in the amplicon sequence data in some of the samples (not detected in 8 samples). The relative abundance reached a maximum of 0.013% in one replicate of sediment from the inter-tidal region (Additional File 4).

### MinION sequencing metagenomics

To evaluate the presence of AMR genes in biofilm and sediment collected from the various sites, samples were sequenced on MinION flow cells to generate long read data. Despite standardised protocols, data production was highly variable, ranging from 9.67 Gb (4,406,214,050 bases) from the sediment collected 100 m east of the STP, to 0.67 Gb (438,604 bases from the sediment from Hangar Cove and 0.18 Gb (58,719,301 bases) from the sediment taken from Back Bay Lagoon. The paucity of data produced from the sediment collected at the two deep marine sites (the Hangar Cove and Back Bay Lagoon samples were both collected at 15 m) was reflected in very short median read lengths (307 bp and 275 bp, respectively) when compared with the samples collected 100 m east of the STP (1,729 bp) (see Additional File 5 for full run statistics, read yield and quality statistics). The aim was to achieve around 3 Gb of data from each site, but this was not always possible, with some sites particularly recalcitrant. Fresh DNA extractions (including the use of large volume extractions on the two 15 m dive collected samples), repeated purifications using AMPure XP beads, including size selection and fresh flow cells, failed to increase the amount of sequence data produced at particular sites (namely the STP intertidal biofilm, Hangar Cove sediment and Back Bay Lagoon sediment samples). There was potentially some undefined contaminant at these sites that may not have been compatible with the flow cell technology. As can be seen in the data presented here, this problem was particularly acute with the 15 m collected sediment samples. However, this issue with flow cell technology has also been noted in other marine samples collected from around the world (Marlon Clark, pers. com.) and is not restricted to Antarctic samples. The variability in output data was reflected in the final polished assemblies. In general, several thousands of contigs were produced from the sequence data produced from each site, ranging from 4,562 contigs from the STP intertidal biofilm samples to 11,604 contigs from the sediment sample collected 100 m east of the STP. Three sites produced much poorer assemblies, namely 1,215 contigs obtained from the biofilm collected 100 m east of the STP; 404 contigs from the Hangar Cove data and only 8 contigs obtained from the Back Bay Lagoon data (Additional File 5). Hence the Back Bay Lagoon data were not included in any further analyses and descriptions. In all sites, circular assemblies (putative plasmids) were produced at between 2 and 5% per site. The number of contigs produced per site was directly reflected in the length of the longest contig which ranged from 2,246,888 bp at the site 100 m east of the STP sediment to only 21,278 bp in the Hangar Cove sediment sample. (Additional File 5). The wide disparity in data generated from each site by this sequencing methodology impacted what was possible in terms of downstream analyses. For example, there was very poor representation of 16 S rDNA genes in the metagenomic assemblies. Therefore, direct comparisons of 16 S rDNA data from the amplicon and metagenomic data, in order to evaluate community composition, was not possible.

### MinION data AMR detection

**Fig. 5:**
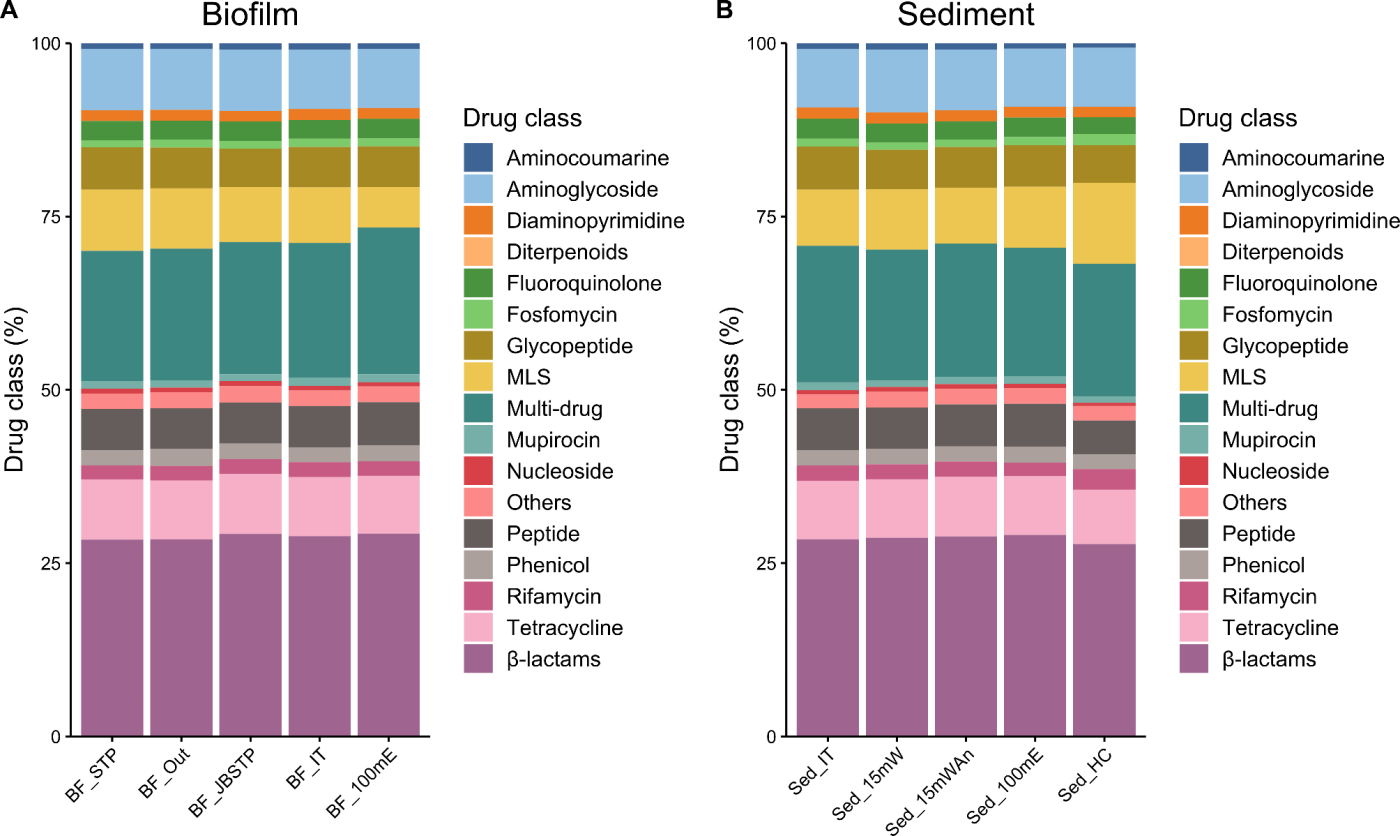
Drug class of ARGs identified at each site (**A**) Biofilm, (**B**) Sediment. Site abbreviations as per Fig. 3

**Fig. 6:**
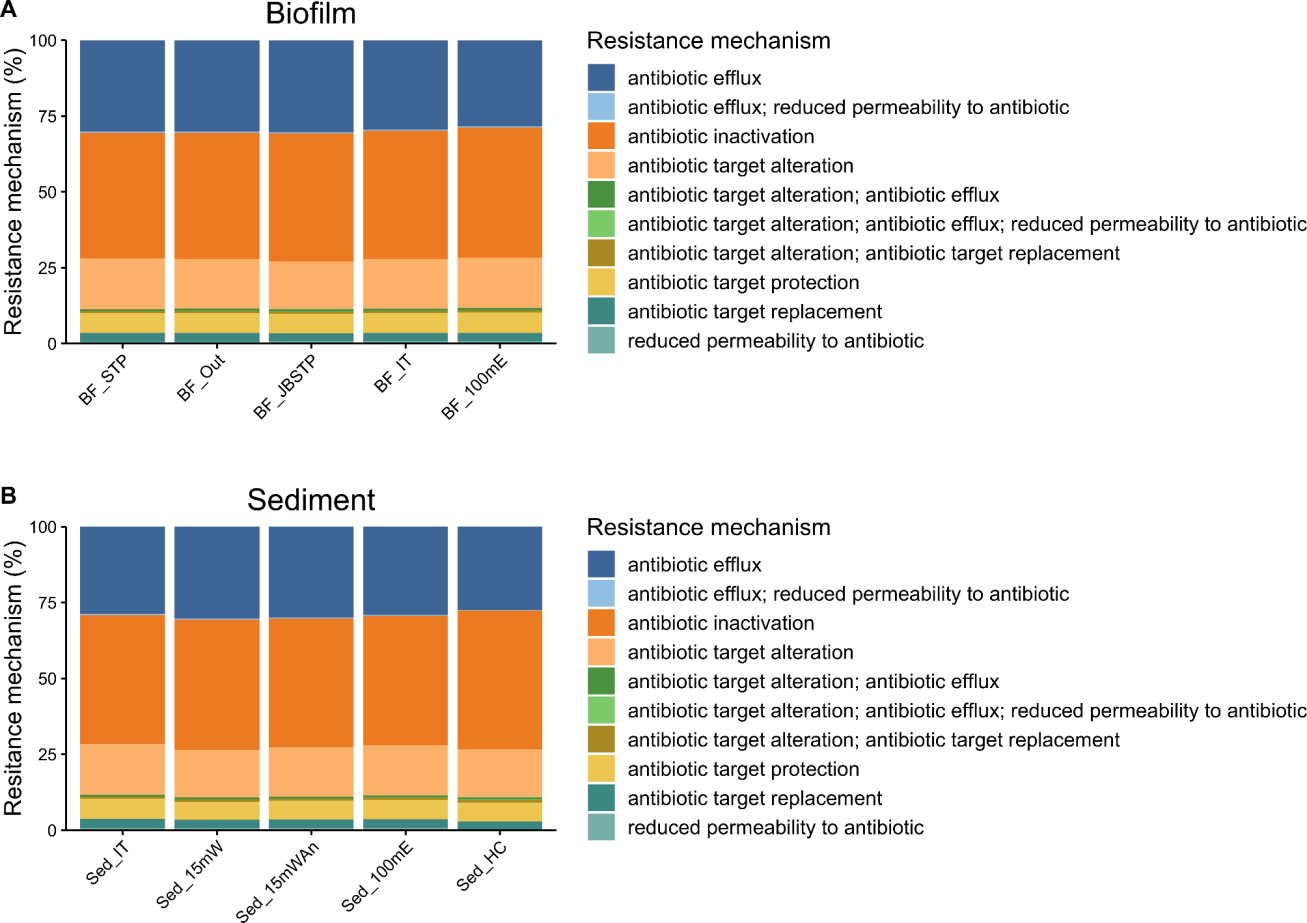
Resistance mechanism of ARGs identified at each site (**A**) Biofilm, (**B**) Sediment. Site abbreviations as per Fig. 3

**Fig. 7:**
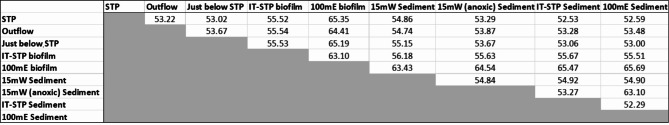
Number of unique ARGs per site. Percentage of unique ARGs between the different sites

The levels of ARG detection using the rgi fargene program and CARD database across the different sites were similar at approximately 1,000 ARGs/Mb, with a range from 877/Mb (biofilm collected 100 m east of the STP) to 1,213/Mb (intertidal sediment near the STP) (Additional File 6). There was no decreasing trend of ARG representation as the sites became more distant to the STP, even if the relatively sparse Hangar Cove data were included. Hangar Cove, although physically close to the STP, is separated from it by the extension of the runway and is subject to different ocean currents (Peck, pers. comm.). The detected ARGs were collated into 14 different drug classes (plus multidrug resistance and “other”) and expressed as percentages to allow comparisons to be made between the highly variable datasets. As observed with the amplicon biodiversity results, there was no significant difference in drug class or resistance mechanism with site, even though sequence coverage across sites varied considerably, as described above (for both drug class and resistance mechanism, ANOVA, *P* ≥ 0.724) (Figs. 5 and 6 and Additional File 6). Given these levels of ARG detection, there was wide representation of individually identified ARGs across the different sites, but approximately 50% of ARGs were unique to each site when pairwise comparisons of ARG composition were made between sites (Fig. 7).

### Antimicrobial sensitivity testing (AST)

This was carried out on the cultivated biofilm and enteric bacterial samples. *Cultivated biofilm bacteria*: EUCAST guidelines were followed for *Pseudomonas* (4 isolates) and there was no phenotypic resistance to any of the tested antibiotics. Other isolates (Table 1) also showed no resistance to any of the tested antibiotics. *Cultivated enteric bacteria*: Out of the 15 enteric bacteria that were analysed, five isolates showed intermediate resistance to pefloxacin, a synthetic broad-spectrum fluoroquinolone, and one isolate was fully resistant to this antibiotic (Additional File 7). Seven isolates were resistant to ampicillin and two showed intermediate resistance to gentamicin. Overall three isolates (*K. pneumoniae* isolate E9, E13 and *E. coli* isolate E6) showed intermediate resistance to more than one clinically relevant antibiotic. (Additional File 7)

## Discussion

These data comprehensively describe the microbial diversity and AMR environment in biofilms and sediments from in and around the STP at Rothera Research Station, Antarctica using a combination of molecular- and culture-based screening techniques. Across all sites, microbial biodiversity is high and associated with an equally diverse resistome (Figs. 3—6). The bacterial phyla are dominated by the Gram-negative *Proteobacteria* and *Bacteroidota* (Fig. 3), consistent with previous Antarctic studies [8, 57]. Large-scale-metagenomics approaches have demonstrated a much higher microbial diversity in Antarctica than previously anticipated, especially with regard to the resistome [8, 23, 57]. The presence of a diverse resistome is expected, as the ability of bacteria to produce antibiotics as part of a natural defence mechanism provides a competitive advantage in this extreme, often nutrient-poor, habitat [7]. Indeed, a metagenomics study on the surface soils of the Mackay Glacier region (South Victoria Land, Antarctica) demonstrated that the number of ARGs in the endemic microbiota is negatively correlated with species richness [8]. Our analyses failed to demonstrate any human impact on the intertidal microbiota around Rothera Point, within the confines of the global analyses conducted. However, they do attest to the complex biodiversity of microbial communities in this remote region.

Previous analyses of the efficacy of wastewater and STPs in Antarctica have largely concentrated on the isolation and culture of sewage microbial markers (e.g., *Escherichia coli or* enterococci) from around the STPs, but more recently with ARG characterisation via PCR [15, 16, 58]. These have shown at least temporary persistence of *E. coli* strains in the water column carrying emergent resistance mechanisms (e.g., strain ST95), ingestion of sewage material by local wildlife and release of antibiotics into the natural environment [15, 16, 58]. The focus on *E. coli* reflects the challenge of distinguishing ‘indigenous’ versus ‘human-introduced’ microbiota. We have a commensurately poor understanding of the natural resistome, the associated ARGs and the compounds they are active against. This is exemplified by the finding of sulphonamide resistance in bacterial DNA extracted from a 1200–1400 YBP ice core [59]. Thus, we cannot assume that all ARGs active against synthetic or semi-synthetic (man-made) antibiotics are a response to human contamination [59].

The surprising result from our site survey was the very poor representation of enteric bacteria in our sequence datasets, even when sampling biofilm directly from the STP. This is likely the result of sample choice. In these experiments we specifically chose to target biofilm and sediment samples as these represent more stable microbial populations rather than the more transient wastewater, which is rapidly diluted as it is expelled from the sewage treatment plant into the surrounding bay. Analysis of these more stable microbial populations is also more likely to provide indication of whether bacteria of human origin (or fragments of DNA generated from such bacteria) are more stably integrated into the endemic communities. There are also other factors which almost certainly influenced the lack of persistence of enteric bacteria. In the STP we sampled from biofilm growing on the tank walls. This is a particular environment, which in retrospect, may be unlikely to be conducive to colonisation by enteric bacteria. The temperature of the treatment tank is ~ 11.7 °C when aerated but increases to ~ 15.9 °C when aeration is periodically turned off (Clark, pers comm from 2023 field season). *E. coli* is mesophilic, growing well between 30 and 42 °C with optimal growth at 37 °C [60]. Growth is impaired below 20 °C and stops at 7.5 °C [61]. Furthermore, an analysis of growth temperatures of a range of faecal and non-faecal coliforms, demonstrated that *E. coli* grows and divides between 20 and 40 °C, which was generally below the growth range of other bacteria such as *Klebsiella pneumoniae* and *Enterobacter aerogenes* [62]. This lack of colonisation and persistence in environmental biofilm due to temperature sensitivity is evidenced in our data by the near absence of enteric bacteria (with a maximum abundance of only 0.013% in one replicate of sediment) and the poor recovery of enteric bacteria from biofilm swabs returned to the UK for isolation. The more resilient temperature tolerances of the non-*E. coli* enteric bacteria, described above, match with the data in this study, where the majority of the cultured enteric bacteria and the annotation of ASVs were allocated to the genus *Klebsiella*. There were only three ASV matches to the genus *Escherichia – Shigella* in the ASV blast annotation, at a maximum of 0.059% in one STP biofilm sample and these data were highly variable between replicates from the same site (Supplemental File S4). In addition, the bacteria cultured from our Antarctic biofilms showed surprising thermal resilience (Table 1) and would likely outcompete the more temperature sensitive enteric bacteria in the wastewater and sewage.

Many additional factors can affect survival of enteric bacteria in Antarctica [63]. In this study, an additional factor for the lack of enteric bacteria in the sequence data may have been the presence of sea water in the STP tank at Rothera Research Station. At the time of the study, due to the expanded population on site and constraints on freshwater production via the desalination plant, sea water (with all its associated marine bacteria) was used to flush all toilets and this salt water passed through the STP. As described above, these marine bacteria appear to be particularly resilient to a wide range of temperatures and also salinity (Hwengwere, pers comm.) and could easily dominate communities under these conditions, outcompeting any *E. coli*. It is well documented that increased salinity deleteriously impacts *E. coli* survival [64], but also a whole variety of environmental factors outside of temperature and salinity affect *E. coli* growth [65]. The biofilms came from a variety of surfaces (STP tank, STP outflow tank, intertidal rocks) and all sediment samples taken near the STP were surface samples. Hence the resident microbiota will also have been subject to the further stresses of desiccation, UV exposure (samples were taken during summer and conditions of 24-hour sunlight), osmotic stress, physical abrasion by sea ice, etc. In particular, the biofilms collected from inside the STP outflow pipe and from the rocks just below, would be subject to relatively long periods of desiccation in between the periodic flushing of wastewater from the STP. These types of conditions all impact bacterial survival [65] and are likely exacerbated by temperature, osmotic shock and other stressors such as high solar radiation when wastewater is ejected into cold Antarctic seawater (at c. -2 to + 1 ^o^C) during the Austral summer. This is evidenced by historic data from Rothera Research Station which tracked survival of viable bacteria at increasing distances from the STP (Additional File 8). However, enteric bacteria can last a considerable number of days in a VNBC state, even in the harsh conditions of the Antarctic [14] and we currently do not know how long their DNAs may remain sufficiently intact in the marine ecosystem to be transferred to other bacteria. We also have no data on the fate of DNA fragments released into the environment when microbial cells die.

Given these hostile conditions, it was unsurprising that when we attempted to culture biofilm and sediment bacteria, the major group isolated was *psychrobacter*, a species which is typically associated with cold environments [66]. Although organisms isolated from polar environments are expected to be stenopsychrophiles, many are europsychrophilic [66]. Indeed, temperature tolerance studies on the cultivated isolates showed that all grew at 22 °C and some survived well at 35 °C (Table 1). Hence, the cultured isolates demonstrated a broad range of growth tolerances and could likely outcompete the more cold- and salt-sensitive enteric bacteria in the biofilms and sediments. In accordance with many previous studies, the culture success of our environmental isolates, compared with total bacterial biodiversity measures, was low at less than 1% (even accounting for the fact that matches of the cultured isolate V3-V4 barcodes were made at 97% sequence identity with the amplicon ASVs, which would potentially include variant strains). None of these (albeit few) cultured isolates showed any sensitivity to 15 common antibiotic classes. In addition, none of the recovered enteric bacteria showed any antibiotic sensitivity of concern according to EUCAST guidelines. These (lack of) sensitivity data are consistent with a previous study from King George Island that also used the disc diffusion assay method [23]. They are further validated by a metagenomic analysis of Antarctic soils [57]. Although this study identified high levels of resistance to clinical antibiotics in Antarctic soils, detailed examination suggested that the resistances identified were of natural origin, not human-driven, emphasizing our lack of knowledge on natural resistomes in Antarctica [55].

In terms of community composition, diversity levels were similar across all sites with no significant differences identified in α diversity and Bray Curtis dissimilarity metrics. These data indicate that there is relatively little effect of a strong feeder source (i.e., STP effluent) indicating that each site has developed a distinct niche community. Despite these metrics, trends were observed in bacterial representation, with *Campilobacterota* dominating the biofilm samples collected at the end of the outflow pipe and their presence was much diminished 100 m away from that site. *Campilobacterota* particularly the genus *Arcobacter*, thrive in diverse habitats. These include seawater but are particularly prevalent in wastewater and around sewage treatment plants [67, 68]. These bacteria can cause human disease, but also play important roles in carbon, nitrogen and sulphur cycling in oceans [69]. The same is true of *Klebsiella* and *Staphylococcus*, which are found in a wide variety of habitats and encompass a range of both human pathogens and environmental strains [9, 70]. Exact assignment of bacteria to species level is challenging using amplicon sequencing because the length of sequence generated is relatively small and thus 100% identity at the sequence level may not be reflected when whole genomes are compared. For example, a strain of *Staphylococcus* isolated from James Ross Island was virtually identical to known strains when comparing 16 S rRNA sequences but was much more divergent at the genome level with less than 85% sequence identity and an inferred DNA hybridization rate of less than 30% and hence was subsequently described as a novel species [9]. Therefore, without sufficient metagenomic data to produce robust metagenome assembled genomes (MAGs) and pangenome data, defining the exact origin and function of the bacteria in this study is difficult and not within scope. What is clear from previous studies is that the Antarctic microbiota will harbour many unique strains and species of bacteria [57].

Regarding the global resistome, our data identified β-lactams as the major drug class, represented by ~ 28% of ARGs, followed by multidrug resistance at ~ 18–20% of ARGs, albeit in this study we designated multidrug resistance as being represented by two or more different drug classes rather than three in other studies [56]. Considerable resistance to β-lactams has been identified in several other studies and is presumed to be a natural defence mechanism against β-lactam producers (e.g., fungi), which are common in the Antarctic environment, especially soils [9, 71]. The next most common drug classes at ~ 8% of ARGs were tetracycline, aminoglycosides and the MLS (macrolides, lincosamides, streptogramin) group, which have also been highlighted as major drug classes in previous studies [8, 23, 57, 71]. The major resistance mechanisms identified were antibiotic inactivation ~ 40% of ARGs, followed by antibiotic efflux ~ 30% ARGs and antibiotic target alteration ~ 16% of ARGs. Previous studies have identified efflux pumps (40% and 60% of ARGs, as reported by [23, 57] respectively) and antibiotic inactivation [57] as major resistance mechanisms. An important point to note is that all previous studies have been carried out on samples from soil, not the marine environment. Therefore, differences in drug class and defence mechanisms may be a result of the particular environment surveyed. Soils are recognised as important reservoirs of AMR [8, 72]. Close physical contact on particulate matter is more likely to drive the requirement for natural defence mechanisms in organism survival rather than a free-living state in the marine environment [73]. Whilst in this study we sampled particulate matter i.e. sediments and biofilm, the interaction with the marine environment could potentially alter interactions and competition between different organisms.

A previous metagenomics analysis of Antarctic microbiota suggested that the transfer of ARGs between species is predominantly vertical with limited horizontal gene transfer [8]. Interestingly, the lowest percentage of circular contigs (i.e. plasmids), a major conduit for horizontal gene transfer in sewage systems [74] was found in the STP biofilm (1.7% of assembled data with higher levels in more distant sites (~ 2–6%)) (Additional File 5). The reason for this relatively low recovery of plasmids in the STP biofilm could be due to sequencing depth, or the nature of the material sampled. A study of psychrophilic and psychrotolerant bacteria identified that many of the isolated plasmids contained genes involved in protection against environmental stresses [75]. Hence the accumulation of higher percentages of plasmids in sites further from the STP may be driven by environmental factors, not AMR.

Detailed interrogation of identified bacterial sequences for analysis of biosynthetic gene clusters and resistance cassettes does require a sufficient level of data to enable the MAGs. Our study emphasised the problematic nature of data uniformity across different sites. Our initial aim was to conduct all Oxford Nanopore MinION sequencing on site, and we aimed to get ~ 3Gb data per site similar to [74]. Unfortunately, despite using kits which included components to combat environmental inhibitors and careful, repeated, purification, the DNA of some samples produced poor yields. Similar types of studies have used a mix of Illumina and Nanopore sequencing [57, 74], which complicates the production of assembles and predicates that at least some sequencing is performed remotely, rather than onsite in real time. The latter may be advantageous, especially when trying to identify which are the critical sites to survey or undertake waste monitoring in real time. Thus, more trials are needed to identify DNA extraction kits more suited to these environmental samples and the production of more uniform data yields.

In addition, although there are studies demonstrating more AMR close to research stations [71, 72, 76], there are no time series to demonstrate how, and if, levels of AMR have accumulated across the years due to human influence. These earlier studies also tended to survey a limited number of ARGs via PCR rather than the more global sequence-based ‘discovery-led’ approaches described here. Sewage disposal is subject to many variables, including a highly seasonally fluctuating, albeit small, human populations, the current flow near any outflow pipes (with sea ice providing a barrier to water mixing and dispersal), the dilution factor of dispersal into the Southern Ocean and the confounding factor of wildlife. For example, huge penguin and seal colonies and their defaecation products are likely to have far more impact on microbial communities than a handful of summer researchers [63]. However, resistance to synthetic antibiotics has been found in filter feeding benthic invertebrates and seals near sewage outflows [16]. In the case of Rothera, elephant seals are often found in close proximity to the STP and presumably are exposed to and may ingest large quantities of water contaminated with faecal microorganisms as a result. Thus, evaluation of AMR in Antarctica needs to be tailored to the characteristics of each environment, whether that is a research station or a more pristine location.

## Conclusions

The increasing application of amplicon and metagenomic approaches to Antarctic microbiota is revealing a highly complex AMR landscape, much of which is natural, a result of the microbial arms war in a nutrient-limited environment. These supposed “cold-adapted” bacteria are far more resilient than previously thought, with some of our cultured Psychrobacter able to grow well at 28 °C and thus liable to succeed in warming seas. Surprisingly our data show little evidence of human enteric bacteria (released in wastewater and sewage) incorporated into more stable biofilm and sediment communities surrounding the sewage treatment plant. This is likely due to a number of factors including sensitivity to the cold, salinity etc. and competition from endemic bacteria. However, we are far from understanding the fate and survivability enteric bacteria in Antarctic waters, or indeed the fate of their DNAs. We currently have no knowledge on how fast this DNA degrades when released into the environment and if it is incorporated into the local environmental microbiota and passed onto wildlife. This requires further research, alongside regular monitoring of the influence of humans on this relatively pristine environment.

## Electronic supplementary material

Below is the link to the electronic supplementary material.

Supplementary Material 1

Supplementary Material 2

Supplementary Material 3

Supplementary Material 4

Supplementary Material 5

Supplementary Material 6

Supplementary Material 7

Supplementary Material 8

## Data Availability

The datasets generated and/or analysed during the current study are available in public depositories, as follows: All V3-V4 molecular barcodes for the cultivated bacteria have been submitted to NCBI BankIt with the accession numbers (OR221159-OR221179 inclusive). The amplicon data have been submitted to NCBI with BioProject ID: PRJNA1042642. The Nanopore data have been submitted to NCBI with accession number BioProject PRJNA1161573 with sequence files for each of the 11 locations (if there are multiple sequence reads for a particular location, these were combined) available as BioSamples with accession numbers SAMN43782255-SAMN43782265 inclusive.
