# Supplementary material for: Assessing the impact of sewage and wastewater on antimicrobial resistance in nearshore Antarctic biofilms and sediments
Source: Environ Microbiome. 2025 Jan 20;20:9. doi: 10.1186/s40793-025-00671-z (PMC11748253; doi:10.1186/s40793-025-00671-z)
Supplement: Supplementary file 1 — Supplementary Material 1 [file 40793_2025_671_MOESM1_ESM.docx]

| **location** | **latitude** | **longitude** |
| --- | --- | --- |
| STP | 67° 34' 04.41997434" S | 068° 07' 27.03872173" W |
| End of outflow pipe | 67° 34' 03.28354546" S | 068° 07' 24.63893895" W |
| Just below outflow pipe | 67° 34' 03.26016016" S | 068° 07' 24.60762686" W |
| Intertidal between pipe and water | 67° 34' 03.10547472" S | 068° 07' 24.43133955" W |
| 100m east of pipe end | 67° 34' 02.88021830" S | 068° 07' 15.99784831" W |
| 15m west of pipe end | 67° 34' 03.05143151" S | 068° 07' 25.67037681" W |
| Hangar Cove | 67° 33' 51.65196925" S | 068° 07' 39.31289867" W |
| Back Bay Lagoon | 67° 35' 42.07134373" S | 068° 15' 12.54959728" W |

**Additional File 1:** Latitude and longitude co-ordinates of the sampled sites. See figures 1 and 2 for further details.
