# Supplementary material for: Assessing the impact of sewage and wastewater on antimicrobial resistance in nearshore Antarctic biofilms and sediments
Source: Environ Microbiome. 2025 Jan 20;20:9. doi: 10.1186/s40793-025-00671-z (PMC11748253; doi:10.1186/s40793-025-00671-z)
Supplement: Supplementary file 2 — Supplementary Material 2 [file 40793_2025_671_MOESM2_ESM.docx]

**Additional File 2:** **List of cultivated bacteria**: Identified at the genus level with percentage identities shown where different isolates have been allocated to the same Class using sequence similarity searching.

1. **Class: Gammproteobacteria**

Thirteen isolates in total from this class.

***Psychrobacter sp.***

Five isolates in this genus varying in sequence identity from 95.8% - 99.3%.

***Pseudomonas sp.***

Four isolates in this genus varying in sequence identity from 94.2% - 98.6%.

***Shewanella sp.***

Three isolates in this genus varying in sequence identity from 96.3% - 97.9%

***Rheinheimera sp.***

Single isolate only.

1. **Class: Flavobacteriia**

***Flavobacterium sp.***

Three isolates in this genus varying in sequence identity from 97.2% - 98.3%

|  | **BF25** | **BF15** | **BF28** |
| --- | --- | --- | --- |
| **BF25** |  | 98.1 | 97.2 |
| **BF15** | 98.1 |  | 98.3 |
| **BF28** | 97.2 | 98.3 |  |

1. **Class: Actinomycetes**

Two isolates from this class, designated as different genera on sequence similarity searching: ***Arthrobactera sp.*** and ***Bracchybacterium sp.*** which share 93.6% sequence identity.

1. **Class: Bacilli**

Two isolates from this class, designated as different genera on sequence similarity searching: ***Trichococcus sp****.* and ***Planococcus sp.*** which share 93.9% sequence identity.

1. **Class: Cytophagia**

***Algoriphagus sp.***

Single isolate only.
