# Supplementary material for: Assessing the impact of sewage and wastewater on antimicrobial resistance in nearshore Antarctic biofilms and sediments
Source: Environ Microbiome. 2025 Jan 20;20:9. doi: 10.1186/s40793-025-00671-z (PMC11748253; doi:10.1186/s40793-025-00671-z)
Supplement: Supplementary file 5 — Supplementary Material 5 [file 40793_2025_671_MOESM5_ESM.docx]

**Additional File 5: Raw assembly and MinION polished assembly metrics for biofilm and sediment samples**

**Raw assembly data: Biofilm samples**

**Raw assembly data: Sediment samples**

**MinION polished assembly data: Biofilm samples**

**
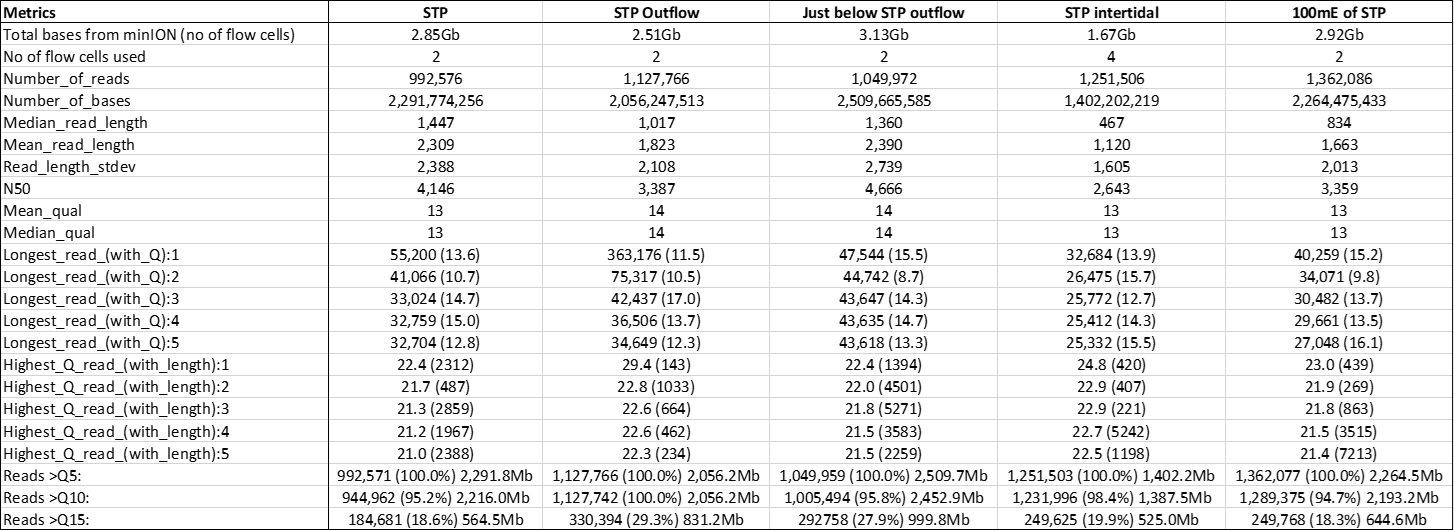
**

**MinION polished assembly data: Sediment samples**
