# Supplementary material for: Assessing the impact of sewage and wastewater on antimicrobial resistance in nearshore Antarctic biofilms and sediments
Source: Environ Microbiome. 2025 Jan 20;20:9. doi: 10.1186/s40793-025-00671-z (PMC11748253; doi:10.1186/s40793-025-00671-z)
Supplement: Supplementary file 6 — Supplementary Material 6 [file 40793_2025_671_MOESM6_ESM.docx]

**Additional File 6:** **ARG metrics**. Number of ARGs per Mb, the number of unique ARGs per site and binning of ARGs into the major drug classes, converted into percentage of total and annotation of ARG resistance mechanism, converted into percentage of total. Data from Hangar Cove is provided, but shaded, as these data do not appear in the main analyses due to paucity of data. These data are provided as a reference to metric in a distant site.
