# Supplementary material for: Assessing the impact of sewage and wastewater on antimicrobial resistance in nearshore Antarctic biofilms and sediments
Source: Environ Microbiome. 2025 Jan 20;20:9. doi: 10.1186/s40793-025-00671-z (PMC11748253; doi:10.1186/s40793-025-00671-z)
Supplement: Supplementary file 7 — Supplementary Material 7 [file 40793_2025_671_MOESM7_ESM.docx]

**Additional File 7: Antibiotic sensitivity profiling of cultured biofilm isolates and enteric bacteria.**

Table 1: Antibiotic susceptibility profiling of biofilm isolates using disk diffusion method with the zone of inhibition measured in mm. *Pseudomonas* isolates with no zone of inhibition (0 mm) are considered intrinsically resistant to the antibiotics tested.

| Isolate No | CIP | C | PEF | CN | FEP | LEV | E | CAZ | CTX | K | TE | MEM |
| --- | --- | --- | --- | --- | --- | --- | --- | --- | --- | --- | --- | --- |
| BF1 | 45 | 0 | 43 | 37 | 38 | 48 | 0 | 39 | 0 | 26 | 23 | 58 |
| BF2 | 32 | 28 | 36 | 24 | 32 | 38 | 24 | 33 | 25 | 28 | 32 | 48 |
| BF3 | 36 | 31 | 32 | 29 | 34 | 30 | 30 | 29 | 28 | 24 | 33 | 43 |
| BF4 | 40 | 43 | 45 | 32 | 44 | 46 | 43 | 44 | 30 | 28 | 32 | 45 |
| BF5 | 38 | 27 | 37 | 28 | 36 | 40 | 42 | 38 | 32 | 28 | 33 | 49 |
| BF6 | 42 | 0 | 38 | 28 | 43 | 37 | 0 | 42 | 0 | 25 | 24 | 50 |
| BF8 | 55 | 28 | 51 | 31 | 43 | 55 | 27 | 32 | 25 | 24 | 36 | 55 |
| BF10 | 55 | 0 | 48 | 24 | 45 | 50 | 0 | 43 | 0 | 26 | 22 | 40 |
| BF13 | 40 | 40 | 42 | 38 | 36 | 39 | 31 | 28 | 36 | 35 | 34 | 44 |
| BF14 | 28 | 33 | 32 | 35 | 40 | 39 | 35 | 23 | 26 | 26 | 33 | 37 |
| BF15 | 51 | 26 | 43 | 34 | 39 | 35 | 31 | 39 | 27 | 27 | 32 | 50 |
| BF17 | 29 | 33 | 24 | 22 | 26 | 26 | 32 | 28 | 26 | 28 | 33 | 36 |
| BF18 | 30 | 40 | 23 | 32 | 35 | 36 | 30 | 36 | 46 | 17 | 26 | 51 |
| BF20 | 36 | 24 | 32 | 24 | 35 | 38 | 22 | 23 | 22 | 28 | 26 | 38 |
| BF21 | 37 | 42 | 33 | 34 | 41 | 37 | 42 | 34 | 32 | 24 | 28 | 53 |
| BF23 | 53 | 0 | 44 | 26 | 34 | 49 | 0 | 35 | 0 | 21 | 22 | 48 |
| BF25 | 43 | 29 | 38 | 31 | 30 | 42 | 40 | 34 | 27 | 28 | 29 | 52 |
| BF26 | 35 | 38 | 33 | 24 | 26 | 40 | 35 | 26 | 24 | 18 | 25 | 48 |
| BF28 | 34 | 34 | 29 | 28 | 24 | 42 | 33 | 30 | 30 | 22 | 23 | 45 |
| BF34 | 32 | 39 | 32 | 32 | 28 | 39 | 31 | 29 | 28 | 28 | 25 | 50 |
| BF35 | 40 | 48 | 45 | 34 | 48 | 52 | 43 | 46 | 22 | 22 | 32 | 45 |

Table 2: Antibiotic susceptibility profiling of enteric isolates using disk diffusion method with the zone of inhibition measured in mm with resistant and intermediate resistance profiles noted as (R) and (I) respectively.

| Isolate no and identities | CN | CIP | LEV | PEF | AMP | FEP | CAZ | CTX | C | MEM |
| --- | --- | --- | --- | --- | --- | --- | --- | --- | --- | --- |
| E1 *(E. coli)* | 21 | 40 | 34 | 22 (I) | 16 | 28 | 22 | 28 | 23 | 27 |
| E2 (*E. coli*) | 16 | 29 | 26 | 26 | 16 | 30 | 22 | 28 | 30 | 26 |
| E3 (*E. coli*) | 16 | 26 | 25 | 25 | 15 | 30 | 21 | 27 | 24 | 25 |
| E4 (*E. coli*) | 16 | 30 | 25 | 23 | 15 | 27 | 21 | 27 | 24 | 25 |
| E5 (*E. coli*) | 17 | 30 | 25 | 25 | 15 | 27 | 21 | 25 | 25 | 25 |
| E6 (*E. coli*) | 15 (I) | 25 | 25 | 22 (I) | 0 (R) | 26 | 25 | 26 | 22 | 22 |
| E7 (*E. coli*) | 16 | 34 | 32 | 24 | 14 | 29 | 22 | 30 | 24 | 25 |
| E8 (*Klebsiella pneumoniae*) | 16 | 25 | 25 | 21 (I) | 0 (R) | 27 | 20 | 27 | 23 | 21 |
| E9 (*Klebsiella pneumoniae*) | 15 (I) | 25 | 24 | 22 (I) | 0 (R) | 25 | 22 | 25 | 24 | 20 |
| E10 (*Enterobacter sp.*) | 16 | 28 | 26 | 26 | 9 (R) | 29 | 24 | 31 | 25 | 25 |
| E11 (*Citrobacter*) | 16 | 28 | 26 | 25 | 20 | 33 | 25 | 30 | 25 | 27 |
| E12 (*Raoultella sp.*) | 16 | 25 | 25 | 22 (I) | 0 (R) | 25 | 20 | 26 | 25 | 20 |
| E13 (*Klebsiella pneumoniae*) | 16 | 21 (I) | 21(I) | 16 (R) | 0 (R) | 30 | 22 | 30 | 24 | 22 |
| E14 (*Klebsiella pneumoniae*) | 16 | 26 | 26 | 24 | 14 | 30 | 22 | 30 | 26 | 24 |
| E15 (*Enterobacter sp.*) | 16 | 34 | 30 | 26 | 0 (R) | 26 | 20 | 27 | 22 | 24 |
