# Supplementary material for: Assessing the impact of sewage and wastewater on antimicrobial resistance in nearshore Antarctic biofilms and sediments
Source: Environ Microbiome. 2025 Jan 20;20:9. doi: 10.1186/s40793-025-00671-z (PMC11748253; doi:10.1186/s40793-025-00671-z)

**Additional File 8: Viability of bacteria in sea water**

These samples were collected by Kevin A Hughes (BAS) and analysed by Claire Evans (National Oceanography Centre) in 2006. Previously unpublished data.

***Flow cytometer enumeration of prokaryotes in seawater***

Seawater samples were collected at 0, 25, 50, 75, 100, 150, 200, 300 and 400 m off shore from the effluent pipe using an inflatable boat. For enumeration of bacteria, 2 ml seawater samples were fixed with a final concentration of 0.5% glutaraldehyde (Sigma Aldrich), before placing at -80°C. Bacterial abundances were determined according to the method of Marie et al. (1999). Briefly, samples were defrosted immediately prior to analysis, diluted with TE-buffer and stained with SYBR-Green I (Molecular Probes) at a final concentration of 1 × 10^-4^ of the commercial stock. Staining was completed in the dark for 10 min at room temperature for 15 min. Analysis was performed on a Becton-Dickinson FACSCalibur flow cytometer and groups were determined in bivariate scatter plots of green fluorescence of stained nucleic acids versus side scatter.

Figure shows the number of bacteria in seawater collected at increasing distance from the outfall, measured using flow cytometry. Counts fell to background levels (i.e., c. 5 × 10^5^ cells ml^-1^; measured in South Cove and near the station wharf, c. 2 km away following the coast, on the other side of Rothera Point) within 50 m of the outfall. Counts of high nucleic acid (HNA) bacteria always exceeded those of low nucleic acid (LNA) bacteria, irrespective of sample collection location.


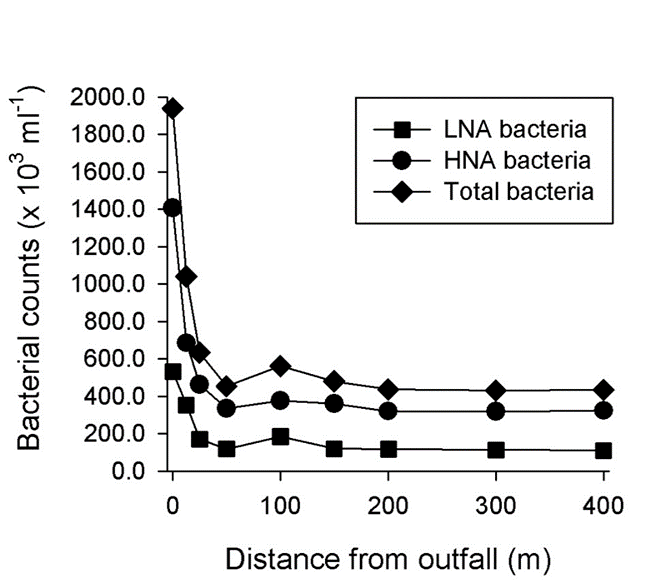

Supplement: Supplementary file 8 — Supplementary Material 8 [file 40793_2025_671_MOESM8_ESM.docx]
